# Comparison of pregnancy outcomes for high morphological scoring mosaic vs. low morphological scoring euploid embryos: a retrospective cohort study

**DOI:** 10.1186/s13048-025-01665-8

**Published:** 2025-04-16

**Authors:** Tangyi Geng, Qiao Zhou, Ying Wang, Hui Ji, Kai Ding, Zichen Zheng, Ye Yang, Junqiang Zhang, Chun Zhao, Xiufeng Ling

**Affiliations:** Department of Reproductive Medicine, The Affliated Obstetrics and Gynaecology Hospital of Nanjing Medical University, Nanjing Women and Children’s Healthcare Hospital, 123 Tianfeixiang, Mochou Road, Nanjing, 210004 China

**Keywords:** PGT-A, Mosaic embryo, Embryo morphological scoring, Pregnancy outcome

## Abstract

**Background:**

Mosaic embryos have been proven to be capable of resulting in live births and have become an option for embryo transfer under certain circumstances. Recent guidelines suggested that embryo morphological scoring should be taken into consideration when selecting mosaic embryos for transfer. Therefore, we introduce a hypothesis that a high morphological scoring mosaic embryo is a better choice compared to a low morphological scoring euploid embryo.

**Materials and Methods:**

This retrospective cohort study included 1641 embryo transfer cycles following next-generation sequencing (NGS)-based preimplantation genetic testing for aneuploidy (PGT-A). Participants were categorized into a mosaic group (87 cycles) and an euploid group (1554 cycles) based on the PGT-A results of the transferred embryos. Statistical methods including multivariate logistic regression analysis and propensity score matching (PSM) were employed to compare the pregnancy outcomes between mosaic and euploid embryo transfer cycles.

**Results:**

Multivariate logistic regression analysis showed that the transfer of mosaic embryos was a prognosis for the reducing live birth rate (*P* = 0.043). Furthermore, when comparing the pregnancy outcomes of the high morphological scoring mosaic embryo transfer group with the low morphological scoring euploid embryo transfer group, no significant differences were observed (*P* > 0.05). Additionally, no significant differences in pregnancy outcomes were found between both the high morphological score low proportion and segmental mosaic group and the low morphological score euploid group (*P* > 0.05).

**Conclusion:**

Our study indicated that morphological scoring has reference value when choosing between euploid and mosaic embryo transfers. Specifically, when the morphological score of euploid embryos is poor, mosaic embryos with high morphological scores could be a viable option after comprehensive prenatal consultation.

**Supplementary Information:**

The online version contains supplementary material available at 10.1186/s13048-025-01665-8.

## Background

With the average age at first marriage increasing year by year, coupled with the impact of environmental pollution, genetics, and other multifactorial influences, it is currently estimated that about 17.5% of couples worldwide are troubled by infertility [1]. Infertility encompasses primary and secondary infertility, with the latter being associated with a higher incidence of adverse obstetric history, approximately 50% resulting from chromosomal abnormalities [2, 3]. With technology advancing rapidly, preimplantation genetic testing for aneuploidy (PGT-A) is extensively utilized to screen for euploid embryos to prevent adverse pregnancy outcomes for patients suffering from single-gene disorders, chromosomal structural rearrangements and recurrent spontaneous abortion (RSA), etc.

The use of PGT-A to screen embryos typically encompasses euploid embryos, mosaic embryos, and aneuploid embryos. A euploid embryo is one that has the correct number of chromosomes, meaning each cell contains 46 chromosomes with 22 pairs and one pair of sex chromosomes (either XX for female or XY for male). On the other hand, an aneuploid embryo has an abnormal number of chromosomes. A mosaic embryo is defined as an embryo containing two or more cell lines with different chromosomal compositions. It is recommended that when PGT-A results include both euploid and mosaic embryos, the transfer of euploid embryos should be prioritized [4]. However, in cases where a patient has transferred a euploid embryo without success and only has mosaic embryos remaining, or in some instances where a patient has only mosaic embryos available, they face the dilemma of whether to undergo another oocyte retrieval cycle or to proceed with the transfer of mosaic embryos. This situation has garnered the attention of clinical physicians. In 2015, following the initial report of healthy infants born following the transfer of mosaic embryos [5], an increasing number of studies are focusing on the live birth rates following the transfer of mosaic embryos [6, 7].

As research into the transplantation of mosaic embryos progresses, it’s found that different types of mosaic embryo transfers yield varying outcomes. For example, younger women [8] undergoing the transfer of mosaic embryos with a lower percentage of mosaicism [9], or those with single or double chromosomal mosaicism, or segmental mosaicism [10], results in better pregnancy outcomes. Moreover, the latest guidelines also suggest that when choosing between euploid and putative mosaic embryos in the low-range for transfer, the testing results should be comprehensively assessed in conjunction with embryonic morphology [11], which aligns with the Preimplantation Genetic Diagnosis International Society (PGDIS) Position Statement on the transfer of mosaic embryos and greatly piques our interest [12]. However, research on the relationship between the morphological scoring of mosaic embryos and pregnancy outcomes is relatively scarce. The embryo morphological scoring is a method for assessing the quality of embryos during in vitro fertilization (IVF). Traditional scoring is often based on visual characteristics of the embryo, including the number of cells, uniformity of cell size, degree of fragmentation, presence of multinucleation, and developmental velocity, which are widely used in clinical practice [13]. Then, we pose the following question: could transferring euploid embryos with low morphological scores result in better pregnancy outcomes compared to transferring mosaic embryos with high morphological scores?

The aim of this study is to explore the pregnancy outcomes following the transfer of mosaic embryos after PGT-A using next-generation sequencing (NGS), as well as comparing the outcomes of transferring high morphological score mosaic embryos with different mosaic proportions and types to those of low morphological score euploid embryos. This information may provide assistance for clinical genetic counseling regarding the transfer of mosaic embryos.

## Materials and methods

### Study population

A retrospective cohort study was conducted in the reproductive center of Nanjing Women and Children’s Healthcare Hospital between March 2016 and January 2024, including 1641 cycles that underwent single-blastocyst transfer following PGT-A. Institutional Review Board approval (2022 KY- 049) was obtained for this study. Patients were assigned into two groups according to the embryo they transfer. Inclusion criteria: ①Age ≥ 20 years and ≤ 45 years; ②Cycles that received NGS-based PGT-A and single-blastocyst transfer. Exclusion criteria: ①Uterine anomalies (unicornuate uterus, bicornuate uterus, didelphys uterus, etc.); ②Endometriosis, adenomyosis, submucous fibroids, endometrial polyps, and other diseases that may affect endometrial receptivity; ③Endocrine and autoimmune diseases. All patients who received mosaic embryo transfer underwent detailed clinical genetic counseling, were informed of the potential clinical risks associated with the transfer, and were advised to undergo prenatal diagnosis.

### PGT-A and frozen-thawed embryo transfer (FET)

Patients underwent controlled ovarian hyperstimulation, oocyte retrieval, and intracytoplasmic sperm injection (ICSI) fertilization, after which embryos were cultured to the blastocyst stage. Blastocyst morphology scoring was based on the Gardner scoring system, and only those with a score of 4BC or higher underwent trophectoderm biopsy followed by freezing. PGT-A was performed for all cycles using next genetic sequencing (NGS). DNA from all samples was amplified through SurePlex DNA Amplification System (Illumina, San Diego, CA, USA). Subsequently, amplified DNA was assessed with a VeriSeq PGS Kit on a the MiSeq system (Illumina, San Diego, CA, USA) according to the manufacturer’s instructions.

After PGT-A, patients who obtained viable euploid embryos or those who, after thorough genetic counseling, chose to transfer mosaic embryos, routinely underwent FET. The first embryo transfer cycle for each patient was included. Endometrial preparation and transfer procedures were chosen according to patients’ characteristics. A Serum HCG test was conducted 14 days after the embryo transfer, and the vaginal ultrasound was done 28 days following the embryo transfer. HCG positivity referred to HCG levels of more than 5 IU/L. Clinical pregnancy was defined as the pregnancy diagnosed via ultrasonographic visualization of gestational sac in the uterus.

### Outcome

In this study, copy number readings from NGS were used to determine mosaicism and obtain the proportion of mosaicism in embryos. Mosaic embryos were identified when the NGS results indicated non-integer copy numbers for a chromosome or chromosome segment, with a mosaic proportion of 30% to 70% [14]. Euploid embryos were designated when the mosaic proportion was < 30%, and aneuploid embryos when the proportion was > 70%. Using a cutoff value of 50% for mosaic proportion based on previous research [7], high-proportion mosaics were defined as > 50%, and low-proportion mosaics as ≤ 50%. Mosaic embryos were further classified into segmental mosaics and whole chromosome mosaics based on the type of chromosomal abnormalities present in the abnormal cell lines.

According to Gardner's scoring system [13], embryos scored 4BC were defined as low morphological scoring, and those ≥ 4BB as high morphological scoring.

Pregnancy outcome indicators were calculated as follows: biochemical pregnancy rate = (number of biochemical pregnancy cycles/number of transfer cycles) × 100%; clinical pregnancy rate = (number of clinical pregnancy cycles/number of transfer cycles) × 100%; live birth rate = (number of live birth cycles/number of transfer cycles) × 100%.

### Data analysis

The study utilized SPSS 27.0 software and R 4.4.0 software for data analysis. Continuous variables are presented as mean and interquartile range (Q1, Q2), with group comparisons made using independent samples t-tests. Categorical variables are expressed as proportions or rates (%), with group comparisons made using chi-square tests or Fisher's exact tests. Multivariate logistic regression analyses were conducted to assess the impact of mosaic embryos on pregnancy outcomes while adjusting for confounding factors. Propensity score matching was performed based on the female age, duration of infertility, basal AMH, and day of embryo development in the high morphological scoring mosaic group with the low morphological scoring euploid embryo transfer cycles. A *P*-value < 0.05 indicated statistically significant differences.

## Results

### Baseline characteristics

A total of 87 mosaic embryo transfer cycles and 1554 euploid embryo transfer cycles were included in this study. The baseline characteristics of the two groups are presented in Table 1. There were no statistically significant differences between the euploid and mosaic groups in terms of male age, body mass index, endometrial preparation protocol ratio, and endometrial thickness (all *P* > 0.05). However, there were statistically significant differences in female age, anti-Mullerian hormone (AMH), and number of days of embryo development (all *P* < 0.05).

**Table 1:** Baseline characteristics of all cycles

Variables	Mosaic embryo transfer cycles	Euploid embryo transfer cycles	*P*
No. of cycles	87	1554	
Maternal age (years)	33.00 (30.50, 38.00)	32.00 (29.00, 35.00)	** < **.001
Parental age (years)	33.00 (30.00, 39.00)	33.00 (30.00, 37.00)	0.190
Endometrial thickness (mm)	8.50 (8.00, 10.00)	8.50 (8.00, 10.00)	0.951
Types of Infertility (%)			0.523
Secondary Infertility	67 (77.01)	1240 (79.85)	
Primary Infertility	20 (22.99)	313 (20.15)	
BMI (kg/m2)	21.90 (20.80, 23.95)	22.00 (20.30, 24.00)	0.731
AMH (ng/ml)	3.23 (1.75, 4.69)	4.00 (2.46, 6.37)	<.001
Embryonic Developmental Days (%)			0.021
D5	34 (39.08)	826 (53.15)	
D6	50 (57.47)	705 (45.37)	
D7	3 (3.45)	23 (1.48)	
Endometrial preparation protocol (%)			0.610
Hormone Replacement Cycles	49 (56.32)	825 (53.09)	
Downregulation + Hormone Replacement Cycles	16 (18.39)	351 (22.59)	
Induced Ovulation Cycles	9 (10.34)	195 (12.55)	
Natural Cycles	13 (14.94)	183 (11.78)	
Biochemical Pregnancy (%)			0.051
YES	56 (64.37)	1148 (73.87)	
NO	31 (35.63)	406 (26.13)	
Clinical Pregnancy (%)			0.462
YES	46 (52.87)	884 (56.89)	
NO	41 (47.13)	670 (43.11)	
Live Birth (%)			0.018
YES	31 (35.63)	756 (48.65)	
NO	56 (64.37)	798 (51.35)	

All continuous data are presented as medians with 25 th and 75 th percentile interquartile ranges (IQR;Q1,Q3)

Abbreviations: *BMI *Body mass index; AMH

### Comparison of pregnancy outcomes between mosaic and euploid embryo transfer cycles

**Fig. 1:**
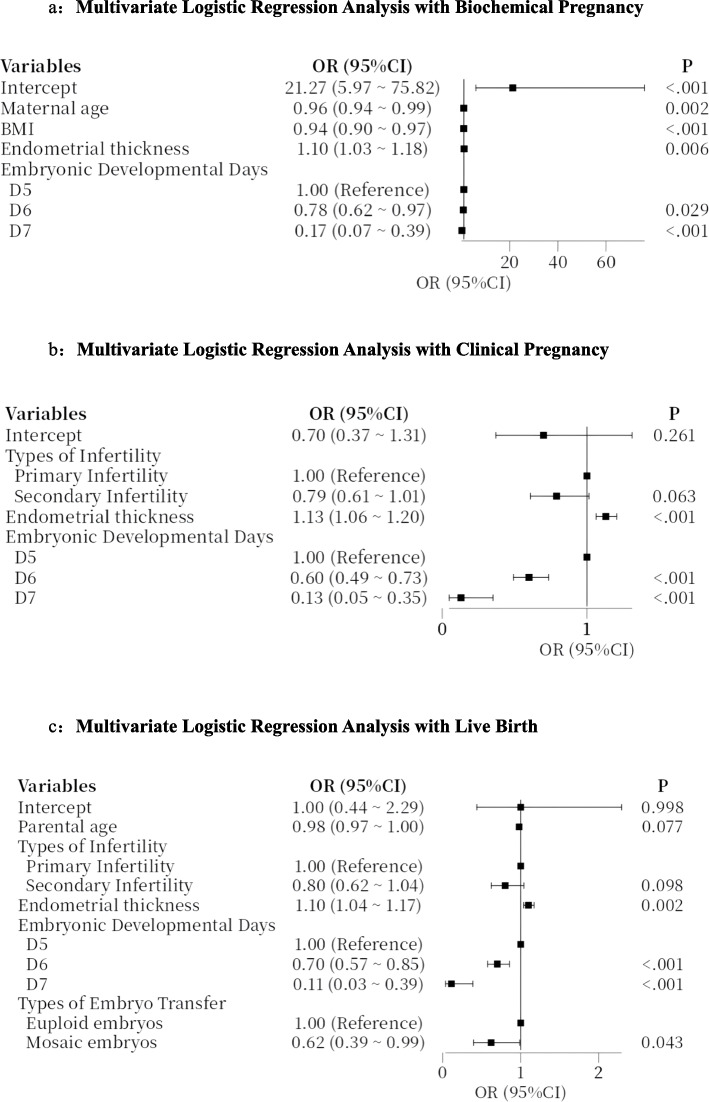
**a** Multivariate Logistic Regression Analysis with Biochemical Pregnancy.** b** Multivariate Logistic Regression Analysis with Clinical Pregnancy. **c** Multivariate Logistic Regression Analysis with Live Birth

Biochemical pregnancy, clinical pregnancy, and live birth were considered the primary outcome measures of this study. Multivariate logistic regression analyses were conducted for each observational indicator and the transfer of euploid embryos served as the reference. The results of the multivariate regression, as depicted in Figs. 1a-c, suggest that the transfer of mosaic embryos is a prognosis for live birth (OR = 0.62, *P* = 0.043), while it is not a prognosis for biochemical pregnancy or clinical pregnancy.

### Comparison of pregnancy outcomes between high morphological score mosaic embryo and low morphological score euploid embryo transfer cycles

A total of 61 cycles were included in the group of high morphological score mosaic embryo transfers, and 366 cycles were included in the group of low morphological score euploid embryo transfers. Propensity score matching (PSM) was performed at a 1:2 ratio based on significant statistical differences in female age from the baseline data. The pregnancy outcomes after matching are shown in Table 2, with no significant statistical differences observed in biochemical pregnancy rate, clinical pregnancy rate, or live birth rate.

**Table 2:** Comparison of pregnancy outcomes between high morphological score mosaic embryo and low morphological score euploid embryo transfer cycles after PSM

Variables	4BC Euploid Embryo transfer cycles	≥ 4BB Mosaic Embryo transfer cycles	*P*
No. of cycles	113	59	
Biochemical Pregnancy (%)			0.498
NO	29 (25.66)	18 (30.51)	
YES	84 (74.34)	41 (69.49)	
Clinical Pregnancy (%)			0.908
NO	47 (41.59)	24 (40.68)	
YES	66 (58.41)	35 (59.32)	
Live Birth (%)			0.154
NO	58 (51.33)	37 (62.71)	
YES	55 (48.67)	22 (37.29)	

### Comparison of pregnancy outcomes between high morphological score low mosaic ratio embryo and low morphological score euploid embryo transfer cycles

A total of 30 cycles were included in the group of high morphological score low mosaic ratio embryos. Propensity score matching at a 1:2 ratio was conducted based on significant statistical differences in endometrial thickness and developmental days compared to the group of low morphological score euploid embryos from the baseline data. The clinical pregnancy rate, biochemical pregnancy rate, and live birth rate after matching for both groups are shown in Table 3, with no significant statistical differences observed.

**Table 3:** Comparison of pregnancy outcomes between high morphological score low mosaic ratio embryo and low morphological score euploid embryo transfer cycles after PSM

Variables	4BC Euploid Embryo transfer cycles	≥ 4BB Low Mosaic Ratio Embryo transfer cycles	*P*
No. of cycles	55	28	
Biochemical Pregnancy (%)			0.242
NO	8 (14.55)	7 (25.00)	
YES	47 (85.45)	21 (75.00)	
Clinical Pregnancy (%)			0.152
NO	10 (18.18)	9 (32.14)	
YES	45 (81.82)	19 (67.86)	
Live Birth (%)			0.232
NO	20 (36.36)	14 (50.00)	
YES	35 (63.64)	14 (50.00)	

### Comparison of pregnancy outcomes between high morphological score segmental mosaic embryos and low morphological score euploid embryos transfer cycles

A total of 54 cycles were included in the group of high morphological score segmental mosaic embryos. Propensity score matching at a 1:2 ratio was performed based on significant statistical differences in female age, baseline anti-Mullerian hormone (AMH), and developmental days compared to the group of low morphological score euploid embryos from the baseline data. The clinical pregnancy rate, biochemical pregnancy rate, and live birth rate after matching for both groups are presented in Table 4, with no significant statistical differences observed.

**Table 4:** Comparison of pregnancy outcomes between high morphological score segmental mosaic embryos and low morphological score euploid embryos transfer cycles after PSM

Variable	4BC Euploid Embryo transfer cycles	≥ 4BB Segmental Mosaic Embryo transfer cycles	*P*
No. of cycles	94	52	
Biochemical Pregnancy (%)			0.203
NO	20 (21.28)	16 (30.77)	
YES	74 (78.72)	36 (69.23)	
Clinical Pregnancy (%)			0.527
NO	33 (35.11)	21 (40.38)	
YES	61 (64.89)	31 (59.62)	
Live Birth (%)			0.071
NO	45 (47.87)	33 (63.46)	
YES	49 (52.13)	19 (36.54)	

## Discussion

In this retrospective analysis, we firstly performed a multivariate logistic regression analysis between the mosaic group and the euploid group, with biochemical pregnancy, clinical pregnancy, and live birth as the main outcome indicators. Our results showed that mosaic embryo transfer is a prognosis for live birth. Subsequently, according to the Gardner grading, we divided the two groups into a high morphological score mosaic subgroup and a low morphological score euploid subgroup. Using PSM based on significant statistical differences in baseline data between the groups, the results showed no significant statistical differences in pregnancy outcomes between these groups. Finally, we divided the high morphological score group into a low proportion mosaic subgroup (with a mosaic ratio ≤ 50%) and a segmental mosaic subgroup based on the type of chromosomal abnormalities carried by the abnormal cell lines in the mosaic embryos. PSM was performed according to the differences in baseline data between these subgroups and the low morphological score euploid group, and the pregnancy outcomes were compared, with no significant statistical differences observed.

Transferring mosaic embryos can indeed result in live births, and there is a consensus on many aspects regarding this. Firstly, compared to the transfer of euploid embryos, the transfer of mosaic embryos might lead to reduced live birth rate [15], although other authors haven’t found any differences [16]. In our study, 46 patients achieved clinical pregnancies after the transfer of mosaic embryos, among which 24 underwent amniocentesis and received fetal chromosomal testing results via telephone follow-up (23 with normal karyotypes and 1 with reported abnormal results), with a total of 31 cycles resulting in live births. It is currently believed that the occurrence of mosaic embryos is mainly due to chromosomal segregation errors during mitosis in normal diploid fertilized eggs, such as delayed chromosome segregation, failure of chromosome separation, chromosome loss, etc. [17, 18]. Studies suggest that these conditions often lead to increased chromosomal instability, which in turn affects pregnancy outcomes [19], which is consistent with the finding of this study that the transfer of mosaic embryos is a prognosis for the reduced live birth rate. Secondly, the transfer of embryos with low proportions or segmental mosaicism often results in better pregnancy outcomes [9], and the most widely accepted threshold is 50% [7, 20]. Furthermore, the 2022 guidelines of the European Society of Human Reproduction and Embryology (ESHRE) state that when only low proportion mosaic embryos (mosaic ratio < 50%) are available for transfer, a new ovarian stimulation cycle is not recommended, and the selection of embryos should be based on a combined assessment of PGT-A results and embryo morphology [11]. Building on this, our study found that the pregnancy outcomes after the transfer of high morphological score low proportion or segmental mosaic embryos were not significantly different from those of low morphological score euploid embryos, suggesting that embryo morphology may have a potential impact on mosaic embryos.

Aneuploid cells play a significant role in the adverse pregnancy outcomes following mosaic embryo transfer [21]**,** and it is believed that aneuploid cells in mosaic embryos are more likely to be distributed in the trophectoderm [22, 23]. In the Gardner scoring system, the score of a blastocyst is based on the comprehensive assessment of the blastocyst development stage, the inner cell mass, and the trophectoderm cells. A multi-regional, multi-center study has shown that transferring blastocysts with low morphological scores results in a reduced live birth rate [24], and when transferring euploid embryos, it has been found that the morphological score of euploid blastocysts is significantly correlated with outcomes such as implantation rate, after adjusting for other confounding factors [25]. These studies suggest that embryos with higher morphological scores often have better developmental potential. Although traditional morphological scoring may not fully reflect the genetic integrity and developmental potential of an embryo, leading to some shortcomings in aspects that are not fully considered in embryos with low scores, methods such as time-lapse imaging systems, various omics technologies (genomics, metabolomics, etc.) for evaluating embryo implantation potential all have varying degrees of limitations or controversies [26, 27]. Hence, a more comprehensive embryo morphology evaluation system needs to be developed, and when making decisions about embryo transfer, it is necessary to consider a variety of biological characteristics and developmental mechanisms, not just based on chromosomal ploidy.

The distribution and morphology of cells within an embryo change significantly as the blastocyst develops, and some recent studies have suggested that changes in biomechanical forces may affect the specialization of cell fate within the embryo, thereby affecting embryonic development. Specifically, the fluid pressure within the blastocoel affects the division pattern of trophectoderm cells, thereby regulating cell differentiation and fate determination [28]; and the fluid shear force within the blastocyst cavity influences the fate determination of the inner cell mass by regulating the expression of Klf2 [29]. The changes in these biomechanical forces are closely related to the expansion of the blastocoel cavity, the quality and quantity of the inner cell mass and trophectoderm cells, and thus further affect the morphological scoring of the blastocyst, which in turn affects the developmental potential of the embryo and ultimately the pregnancy outcome. Our study found no significant difference in pregnancy outcomes after transfer between high morphological score mosaic embryos and low-scoring euploid embryos. Based on the aforementioned research, we speculate that the high morphological scoring of low proportion mosaic embryos, due to more suitable biomechanical forces, may to some extent compensate for the insufficient developmental potential of mosaic embryos.

To sum up, personalized treatment is particularly important during IVF [30]. Given the complexity of mosaic embryos and the potential impact of various factors on pregnancy outcomes, personalized consideration is essential in deciding whether to transfer mosaic embryos. This includes not only genetic testing results but also embryo morphology, patient age, and financial status [31], etc.

Our study is the first to explore whether there is a difference in pregnancy outcomes after the transfer of high morphological score mosaic embryos and low morphological score euploid embryos, and further indicates that the morphological scoring of embryos has some reference value when deciding whether to transfer mosaic embryos. However, this study still has the following limitations. Firstly, this study is a single-center retrospective study, and the sample size is relatively small compared to similar types of studies. Multi-center, larger sample size randomized controlled trials with long-term follow-up are needed to further verify the findings. Secondly, most reproductive centers, including ours, currently perform PGT-A using trophectoderm cell biopsy, and many studies have found that there is a partial inconsistency between the genetic status of trophectoderm cell biopsy and the inner cell mass [32, 33]. The diagnostic accuracy and technical limitations of PGT-A may affect the diagnosis of mosaic embryos. Thirdly, although embryo morphological scoring in our reproductive center is conducted by embryologists with extensive experience, the study is retrospective in nature, and the evaluation was carried out by multiple embryologists, inevitably introducing subjective differences among them. In addition, more in-depth research is needed on the mechanism of live birth after mosaic embryo transfer to provide more objective assessment basis for the transfer value of mosaic embryos.

## Conclusion

Our study found that the transfer of mosaic embryos is a prognosis for the reduction of live birth rates, and there is no significant difference in pregnancy outcomes after the transfer of high morphological score mosaic embryos compared to low morphological score euploid embryos. This suggests the reference value of morphological scoring when choosing between euploid and mosaic embryo transfers, especially when the morphological score of euploid embryos is poor, high-scoring mosaic embryos may also be an option.

## Supplementary Information

Supplementary Material 1.

## Data Availability

The data that support the findings of this study are available from the corresponding author upon reasonable request.
