# Supplementary material for: Comparison of pregnancy outcomes for high morphological scoring mosaic vs. low morphological scoring euploid embryos: a retrospective cohort study
Source: J Ovarian Res. 2025 Apr 16;18:79. doi: 10.1186/s13048-025-01665-8 (PMC12004691; doi:10.1186/s13048-025-01665-8)
Supplement: Supplementary file 1 — Supplementary Material 1. [file 13048_2025_1665_MOESM1_ESM.docx]

| Variables | 4BC Euploid Embryo transfer cycles | ≥4BB Low Mosaic Ratio  Embryo transfer cycles | *P* |
| --- | --- | --- | --- |
| No. of cycles | 366 | 30 |  |
| Maternal age (years) | 31.00 (29.00, 34.00) | 31.00 (29.00, 34.00) | 0.058 |
| Parental age (years) | 32.00 (29.00, 36.00) | 32.00 (30.00, 35.75) | 0.753 |
| Endometrial thickness (mm) | 8.50 (8.00, 10.00) | 9.00 (8.00, 11.00) | 0.048 |
| Types of Infertility (%) |  |  | 0.572 |
| Secondary Infertility | 263 (71.86) | 23 (76.67) |  |
| Primary Infertility | 103 (28.14) | 7 (23.33) |  |
| BMI (㎏/m2) | 21.90 (20.30, 24.20) | 22.00 (19.88, 24.50) | 0.739 |
| AMH (ng/ml) | 4.12 (2.31, 6.67) | 4.17 (2.11, 5.09) | 0.220 |
| Embryonic Developmental Days (%) |  |  | 0.002 |
| D5 | 81 (22.13) | 15 (50.00) |  |
| D6 | 265 (72.40) | 15 (50.00) |  |
| D7 | 20 (5.46) | 0 (0.00) |  |
| Endometrial preparation protocol (%) |  |  | 0.241 |
| Hormone Replacement Cycles | 194 (53.01) | 14 (46.67) |  |
| Downregulation + Hormone Replacement Cycles | 87 (23.77) | 5 (16.67) |  |
| Induced Ovulation Cycles | 44 (12.02) | 4 (13.33) |  |
| Natural Cycles | 41 (11.20) | 7 (23.33) |  |

Supplementary table1: **Baseline Characteristics of High Morphological Score Low Mosaic Ratio Embryo and Low Morphological Score Euploid Embryo transfer cycles**

All continuous data are presented as medians with 25th and 75th percentile interquartile ranges (IQR;Q1,Q3).

Supplementary table2: **Baseline Characteristics of High Morphological Score Segmental Mosaic Embryos and Low Morphological Score Euploid Embryos transfer cycles**

| Variables | 4BC Euploid Embryo transfer cycles | ≥4BB Segmental  Embryo transfer cycles | *P* |
| --- | --- | --- | --- |
| No. of cycles | 366 | 54 |  |
| Maternal age (years) | 31.00 (29.00, 34.00) | 33.50 (31.00, 37.00) | <.001 |
| Parental age (years) | 32.00 (29.00, 36.00) | 32.00 (31.00, 37.00) | 0.235 |
| Endometrial thickness (mm) | 8.50 (8.00, 10.00) | 9.00 (8.00, 10.00) | 0.409 |
| Types of Infertility (%) |  |  | 0.362 |
| Secondary Infertility | 263 (71.86) | 42 (77.78) |  |
| Primary Infertility | 103 (28.14) | 12 (22.22) |  |
| BMI (㎏/m2) | 21.90 (20.30, 24.20) | 21.60 (20.72, 23.85) | 0.775 |
| AMH (ng/ml) | 4.12 (2.31, 6.67) | 3.46 (2.11, 4.69) | 0.046 |
| Embryonic Developmental Days (%) |  |  | <.001 |
| D5 | 81 (22.13) | 27 (50.00) |  |
| D6 | 265 (72.40) | 26 (48.15) |  |
| D7 | 20 (5.46) | 1 (1.85) |  |
| Endometrial preparation protocol (%) |  |  | 0.400 |
| Hormone Replacement Cycles | 194 (53.01) | 29 (53.70) |  |
| Downregulation + Hormone Replacement Cycles | 87 (23.77) | 10 (18.52) |  |
| Induced Ovulation Cycles | 44 (12.02) | 5 (9.26) |  |
| Natural Cycles | 41 (11.20) | 10 (18.52) |  |

All continuous data are presented as medians with 25th and 75th percentile interquartile ranges (IQR;Q1,Q3).

Supplementary table3: **Baseline Characteristics of High and Low Morphological Score Segmental Mosaic Embryo transfer cycles**

| Variables | 4BC Segmental Mosaic Embryo transfer cycles | ≥4BB Segmental Mosaic  Embryo transfer cycles | *P* |
| --- | --- | --- | --- |
| No. of cycles | 27 | 54 |  |
| Maternal age (years) | 33.00 (30.00, 38.50) | 33.50 (31.00, 37.00) | 0.864 |
| Parental age (years) | 33.00 (30.00, 39.50) | 32.00 (31.00, 37.00) | 0.732 |
| Endometrial thickness (mm) | 8.00 (8.00, 9.50) | 9.00 (8.00, 10.00) | 0.257 |
| Types of Infertility (%) |  |  | 0.466 |
| Secondary Infertility | 19 (70.37) | 42 (77.78) |  |
| Primary Infertility | 8 (29.63) | 12 (22.22) |  |
| BMI (㎏/m2) | 22.80 (20.62, 24.07) | 21.60 (20.72, 23.85) | 0.674 |
| AMH (ng/ml) | 2.28 (1.08, 4.04) | 3.46 (2.11, 4.69) | 0.099 |
| Embryonic Developmental Days (%) |  |  | 0.009 |
| D5 | 5 (18.52) | 27 (50.00) |  |
| D6 | 20 (74.07) | 26 (48.15) |  |
| D7 | 2 (7.41) | 1 (1.85) |  |
| Endometrial preparation protocol (%) |  |  | 0.652 |
| Hormone Replacement Cycles | 17 (62.96) | 29 (53.70) |  |
| Downregulation + Hormone Replacement Cycles | 5 (18.52) | 10 (18.52) |  |
| Induced Ovulation Cycles | 3 (11.11) | 5 (9.26) |  |
| Natural Cycles | 2 (7.41) | 10 (18.52) |  |

All continuous data are presented as medians with 25th and 75th percentile interquartile ranges (IQR;Q1,Q3).

Supplementary table4: **Comparison of Pregnancy Outcomes between Low and High Morphological Score Segmental Mosaic Embryo transfer cycles after 1:1 PSM**

| Variables | 4BC Segmental Mosaic Embryo transfer cycles | ≥4BB Segmental Mosaic  Embryo transfer cycles | *P* |  |
| --- | --- | --- | --- | --- |
|  |  |  |  |  |
| No. of cycles | 26 | 26 |  |  |
| Biochemical Pregnancy (%) |  |  | 0.080 |  |
| NO | 12 (46.15) | 6 (23.08) |  |  |
| YES | 14 (53.85) | 20 (76.92) |  |  |
| Clinical Pregnancy (%) |  |  | 0.096 |  |
| NO | 16 (61.54) | 10 (38.46) |  |  |
| YES | 10 (38.46) | 16 (61.54) |  |  |
| Live Birth (%) |  |  | 0.768 |  |
| NO | 18 (69.23)) | 17 (65.38) |  |  |
| YES | 8 (30.77) | 9 (34.62) |  |  |

Supplementary table5: **Baseline Characteristics and Pregnancy Outcomes of High and Low Morphological Score Low Mosaic Ratio Embryo transfer cycles**

| Variables | 4BC Low Mosaic Ratio Embryo transfer cycles | ≥4BB Low Mosaic Ratio  Embryo transfer cycles | *P* |
| --- | --- | --- | --- |
| No. of cycles | 14 | 30 |  |
| Maternal age (years) | 32.00 (28.25, 38.00) | 32.50 (30.25, 36.00) | 0.869 |
| Parental age (years) | 31.50 (30.00, 39.50) | 32.00 (30.00, 35.75) | 0.713 |
| Endometrial thickness (mm) | 9.00 (8.00, 10.00) | 9.00 (8.00, 11.00) | 0.407 |
| Types of Infertility (%) |  |  | 0.620 |
| Secondary Infertility | 9 (64.29) | 23 (76.67) |  |
| Primary Infertility | 5 (35.71) | 7 (23.33) |  |
| BMI (㎏/m2) | 21.65 (20.58, 23.90)) | 22.00 (19.88, 24.50) | 0.659 |
| AMH (ng/ml) | 2.29 (1.27, 3.55) | 4.17 (2.11, 5.09) | 0.174 |
| Embryonic Developmental Days (%) |  |  | 0.173 |
| D5 | 4 (28.57) | 15 (50.00) |  |
| D6 | 9 (64.29) | 15 (50.00) |  |
| D7 | 1 (7.14) | 0 (0.00) |  |
| Endometrial preparation protocol (%) |  |  | 0.632 |
| Hormone Replacement Cycles | 9 (64.29) | 14 (46.67) |  |
| Downregulation + Hormone Replacement Cycles | 2 (14.29) | 5 (16.67) |  |
| Induced Ovulation Cycles | 2 (14.29) | 4 (13.33) |  |
| Natural Cycles | 1 (7.14) | 7 (23.33) |  |
| Biochemical Pregnancy (%) |  |  | 0.155 |
| NO | 7 (50.00) | 7 (23.33) |  |
| YES | 7 (50.00) | 23 (76.67) |  |
| Clinical Pregnancy (%) |  |  | 0.085 |
| NO | 8 (57.14) | 9 (30.00) |  |
| YES | 6 (42.86) | 21 (70.00) |  |
| Live Birth (%) |  |  | 0.181 |
| NO | 10 (71.43) | 15 (50.00) |  |
| YES | 4 (28.57) | 15 (50.00) |  |

All continuous data are presented as medians with 25th and 75th percentile interquartile ranges (IQR;Q1,Q3).
